# Laboratory-based study of drug resistance and genotypic profile of multidrug-resistant tuberculosis isolates in Salvador, Bahia, Brazil

**DOI:** 10.1590/0037-8682-0013-2022

**Published:** 2022-07-25

**Authors:** Erivelton de Oliveira Sousa, Rita Terezinha de Oliveira Carneiro, Fátima Cristina Onofre Fandinho Montes, Emilyn Costa Conceição, Patricia Bartholomay, Jamocyr Moura Marinho, Karla Valéria Batista Lima, Marcio Santos da Natividade, Wildo Navegantes de Araújo, Eliana Dias Matos, Theolis Barbosa

**Affiliations:** 1 Laboratório Central de Saúde Pública Professor Gonçalo Moniz (LACEN-BA), Salvador, BA, Brasil.; 2 Fundação Oswaldo Cruz, Instituto de Pesquisa Gonçalo Moniz, Salvador, BA, Brasil.; 3 Fundação Oswaldo Cruz, Centro de Referência Professor Hélio Fraga, Laboratório de Bacteriologia da Tuberculose, Rio de Janeiro, RJ, Brasil.; 4 Universidade Federal do Rio de Janeiro, Instituto de Microbiologia Paulo de Góes, Rio de Janeiro, RJ, Brasil.; 5 Universidade de Brasília, Brasília, DF, Brasil.; 6 Escola Baiana de Medicina e Saúde Pública, Faculdade de Medicina, Salvador, Bahia, Brasil.; 7 Instituto Evandro Chagas, Seção de Bacteriologia e Micologia, Ananindeua, Pará, Brasil.; 8 Universidade Federal da Bahia, Instituto de Saúde Coletiva, Salvador, Bahia, Brasil.; 9 Rede Brasileira de Pesquisa em Tuberculose, Rio de Janeiro, RJ, Brasil.

**Keywords:** Tuberculosis, Molecular epidemiology, Drug resistance, Genotyping

## Abstract

**Background:**

Surveillance of multidrug resistant/extensively drug-resistant tuberculosis (MDR/XDR-TB) is essential to guide disease dissemination control measures. Brazil contributes to a significant fraction of tuberculosis (TB) cases worldwide, but only few reports addressed MDR/XDR-TB in the country.

**Methods:**

This cross-sectional, laboratory-based study describes the phenotypic resistance profiles of isolates obtained between January 2008 and December 2011 in Bahia, Brazil, and sociodemographic, epidemiological, and clinical characteristics (obtained from mandatory national registries) of the corresponding 204 MDR/XDR-TB patients. We analyzed the mycobacterial spoligotyping and variable number of tandem repeats of mycobacterial interspersed repetitive units in 12-loci profiles obtained from Salvador.

**Results:**

MDR/XDR-TB patients were predominantly male, had a median age of 43 years, belonged to black ethnicity, and failed treatment before MDR-TB diagnosis. Nearly one-third of the isolates had phenotypic resistance (evaluated by mycobacteria growth indicator tube assay) to second-line anti-TB drugs (64/204, 31%), of which 22% cases (14/64) were diagnosed as XDR-TB. Death was a frequent outcome among these individuals and was associated with resistance to second-line anti-TB drugs. Most isolates successfully genotyped belonged to the Latin-American Mediterranean (LAM) Family, with an unprecedented high proportion of LAM10-Cameroon subfamily bacilli. More than half of these isolates were assigned to a unique cluster by the genotyping methods performed. Large clusters of identical genotypes were also observed among LAM SIT42 and SIT376 strains.

**Conclusions:**

We highlight the need for strengthening local and national efforts to perform early detection of TB drug resistance and to prevent treatment discontinuation to limit the emergence of drug-resistant strains.

## INTRODUCTION

Tuberculosis (TB) causes notable infectious disease morbimortality worldwide. In 2019, 10 million new TB cases and 1.4 million deaths were estimated, although this disease is considered highly curable^1^. The prevalence of drug-resistant TB forms has gradually increased owing to accumulation in mutations that interfere with the action of anti-TB drugs. This phenomenon threatens to derail the current goal of eliminating this disease by 2035, especially in the light of the observed hindrances in TB patient care resulting from the coronavirus disease pandemic, since 2020, in middle- and low-income settings^2^. 

TB strains presenting combined resistance to at least the main first-line anti-TB drugs, rifampicin (R) and isoniazid (H), are termed multidrug-resistant (MDR). MDR-TB therapy entails a longer, more aggressive, and more expensive treatment course involving the use of second-line drugs associated with more frequent and severe side effects as well as higher rates of death and disability ^2^ ^,^ ^3^. 

MDR-TB forms presenting additional resistance to fluoroquinolone combined with an injectable second-line anti-TB drug, termed extensively drug-resistant TB (XDR-TB), are associated with an even poorer prognosis and more challenging therapeutic management^3^. The emergence of MDR/XDR-TB is associated with inadequate disease treatment, mostly secondary to multiple, irregular, and/or incomplete treatment regimens^4^. Treatment strategies adopted without prior knowledge of strain resistance to anti-TB drugs contribute to treatment failure and the development of further resistance^5^ ^,^ ^6^. Primary MDR/XDR-TB transmission is increasingly reported in endemic regions, further aggravating this scenario^6^. 

Brazil reported 35.0 TB cases/100,000 inhabitants in 2019. Salvador (Bahia) reported 49.4 cases and 2.6 deaths/100,000 inhabitants in the same year, ranking fifth among the Brazilian state capitals^7^. A state reference hospital-based study performed from July 2001 to July 2003 was the first attempt to estimate the prevalence of MDR-TB resistance in Salvador; 14% of all TB cases diagnosed were classified as MDR-TB^8^. The present study was conducted to characterize the MDR-TB cases in Salvador, regarding resistance to second-line anti-TB drugs, and the sociodemographic, epidemiologic, and clinical characteristics of the affected patients. 

## MATERIAL AND METHODS

### Study setting

The Professor Goncalo Moniz Public Health Central Laboratory (LACEN-BA) is the reference diagnostic laboratory for Bahia, northeastern Brazil. This service maintains a collection of drug-resistant isolates obtained for phenotypic testing of first-line anti-TB drug resistance, performed for all patients from the state reference institution for the treatment of complicated TB cases (Octavio Mangabeira Hospital). Until recently, this hospital was the only healthcare unit responsible for providing care to patients diagnosed with MDR-TB. Our study focused exclusively on MDR-TB isolates.

### Culturing of isolates and phenotypic anti-TB drug resistance testing

*M. tuberculosis* isolates (N=392) obtained from January 2008 to December 2011 were considered for analysis. Biochemical species identification, susceptibility to first-line anti-TB drugs (R, H, ethambutol (E)), and resistance to streptomycin (S) had been established before storage. Mycobacterial species was determined by culturing in a Lowenstein-Jensen (LJ) solid medium containing p-nitrobenzoic acid (500 µg/ml) and tiophene-2-carboxilic acid hydrazide (2 µg/ml), a niacin test, and phenotypic colony characteristics^9^.

Drug sensitivity to H, R, E, and S was determined according to the proportions’ method established by Canetti^10^ at concentrations of 0.2, 40, 2, and 4 µg/ml, respectively. All isolates from the LACEN-BA bacteria archive were kept frozen at -20°C in a Middlebrook 7H9 medium supplemented with glycerol. Multiple isolates were sometimes obtained by serial testing (13%, N=52/392). Before analysis, the isolates were expanded in LJ solid medium tubes, and isolates that did not grow under the culturing conditions (34%; 134/392) were excluded. We analyzed the solely available or the most recently obtained isolate from each patient, resulting in 204 analyzed isolates.

Phenotypic drug sensitivity testing of first- and second-line anti-TB drug resistance was performed in the National Reference Laboratory, Professor Hélio Fraga Reference Center (CRPHF/ENSP-FIOCRUZ), Rio de Janeiro/Brazil using the BACTEC MGIT960 automated liquid culture system (Becton Dickinson Diagnostic Systems, MD). All first-line anti-TB drugs previously tested and the second-line anti-TB drugs ofloxacin (Ofx), kanamycin (Km), amikacin (Am), and capreomycin (Cm) were assessed. Isolates with discordant susceptibility to the first-line anti-TB drugs when comparing solid culture and liquid culture test results were then retested by liquid culture; these results were considered the final data. 

### Sociodemographic, epidemiologic, and clinical data

Standardized sociodemographic, epidemiologic, and clinical information on MDR-TB patients was collected from governmental compulsory notification databases: SINAN (http://portalsinan.saude.gov.br/), the national registry for mandatory notifiable diseases^11^, and SITE-TB (http://sitetb.saude.gov.br/), the national registry for the application of special TB therapy regimens^12^. Additional information regarding reported contacts and treatment outcomes before 2012 and information missing from the compulsory notification databases were obtained from the reference hospital medical records. 

The variables analyzed were sex; age; self-reported race/ethnicity^13^; home address; years of schooling; drugs used in previous TB treatment(s), treatment period and outcome - defaulted from therapy, failure, cure, death; known contact with an MDR-TB patient; primary or secondary resistance notification; reported comorbidities, including human immunodeficiency virus (HIV) test status and results, diabetes, mental illness(es), drug and/or alcohol use; extent of pulmonary disease; duration and outcome of the treatment administered at the time of isolate obtainment.

This study complied with the ethical principles expressed in the Helsinki Declaration and the Brazilian legislation in force during sample and data collection (Resolution 196/96)^14^. This investigation was approved by the Institutional Review Board of Gonçalo Moniz Research Center (CEP-CPqGM/FIOCRUZ) under CAAE registry no. 0006.0.225.000-11.

### Georeferencing using home address information

Spatial distribution of the MDR-TB cases considered the 417 municipalities comprising the territory of Bahia. MDR-TB cases were georeferenced based on residential addresses using Google Earth Pro software. MDR-TB cases were plotted in a shape file containing the vectorized political map of Bahia, provided by the Brazilian Institute of Geography and Statistics, using ArqGis 2.18 software. The municipalities wherein MDR-TB cases were notified during the study period were highlighted according to the number of occurrences (strata considered: 0, 1, or ≥2 occurrences). XDR-TB cases were marked using the coordinates corresponding to the geometric center of each municipality.

### DNA extraction and genotypic characterization of isolates

DNA was obtained from one to two loops of bacterial mass grown in a duplicate tube containing LJ medium as described above, according to the protocol described by Van Embden^15^. Spoligotyping was performed following the protocol published by Cowan^16^. Cultures of H37Rv *M. tuberculosi*s and *M. bovis* reference strains were used as positive controls. Positivity patterns for each of the 43 alleles were compared with the reference patterns available in the SITVIT2 database (http://www.pasteur-guadeloupe.fr:8081/SITVIT2/)^17^, enabling the identification of Mycobacterial lineages, sublineages, and Shared International Types (SITs).

In parallel, MIRU-VNTR 12-loci typing was performed using previously extracted DNA, according to Supply^18^ and Kremer^19^. The 12 loci investigated were as follows: MIRU02, MIRU04, MIRU40, MIRU10, MIRU16, MIRU20, MIRU23, MIRU24, MIRU26, MIRU27, MIRU31, and MIRU39. Negative and positive controls were used as described above. The molecular weight marker LIZ1200 (GeneScanTM LIZ1200®, Applied Biosystems) was added to each product. Capillary electrophoresis was performed using an ABI 3130 sequencing platform (Applied Biosystems, Foster City, EUA). GeneMapper 4.0 (Applied Biosystems) software was used for fragment analysis. The MIRU-VNTRplus analysis tool (https://www.miru-vntrplus.org/MIRU/index.faces) was used for tree-based lineage identification by similarity (constructed using the unweighted pair group method with arithmetic mean-UPGMA algorithm) and to obtain a dendrogram of the XDR-TB isolates.

### Statistical analyses

Categorical variables are described as absolute and relative frequencies. Mean, standard deviation, and 95% confidence interval (95% CI) were reported for quantitative variables with normal distribution. Median and interquartile range were reported for non-normally distributed variables. Proportions were compared using the chi-square test and prevalence ratio. Central measures were compared using Student’s t-test for parametric variables and the Mann-Whitney test for non-parametric variables. All tests considered an alpha error of 5% to distinguish significant differences. We used the STROBE cross-sectional reporting guidelines to ensure proper data reporting^20^.

## RESULTS

Approximately two-thirds of the 204 MDR-TB isolates (66%; 135/204) were resistant to anti-TB drugs other than R and H; additional resistance to both E and S was observed (40%; 55/135). Over one-third of the isolates (37%; 50/135) were resistant to at least one second-line anti-TB drug and were therefore considered pre-XDR-TB patients^21^. Seven percent of patients (14/204) were classified as XDR-TB (two isolates were resistant to both ofloxacin and capreomycin; one isolate was resistant to ofloxacin, kanamycin, and amikacin; and 11 isolates were resistant to ofloxacin, capreomycin, kanamycin, and amikacin).

In all groups, most patients were male (65%; 132/204), and most of them belonged to black ethnicity (African-Brazilian and mixed-race: 88%; 148/169). A small proportion of patients was formally employed (22%; 38/169), and most patients reported a maximum of 7 years of primary education (60%; 80/132). Family income was not registered in the notification databases or on medical records. Other sociodemographic and epidemiologic characteristics of the patients are listed in Table 1. Median age (approximately 40 years) was similar for the MDR-TB, pre-XDR-TB, and XDR-TB groups. Patient medical records frequently showed previous treatment failure and default. While the frequency of intermittent treatment was not different among the groups, patients with strains resistant to at least one second-line anti-TB drug failed treatment more often than the other MDR-TB patients.

**TABLE 1:** Sociodemographic characteristics of MDR-TB cases from Bahia, Brazil, with viable isolates obtained (2008-2011).

Variables	Strains susceptible to all second-line anti-TB drugs^1^ MDR-TB (n=140)	Strains resistant to at least one second-line anti-TB drug^2^		Total (n=204)	Patients with resistant^2^ vs. susceptible^1^ strains Prevalence ratio [95% CI]
		Pre-XDR-TB (n=50)	XDR-TB (n=14)		
Male	72% (101/140)	58% (29/50)	71% (10/14)	65% (132/204)	0.84 [0.68-1.05]
Age [min.-max.]^3^	36 [18-71]	39 [18-65]	43 [20-72]	38 [18-72]	
**Comorbidities** ^4^					2.62 [1.79-3.85]***
Not reported	52% (73/140)	40% (20/50)	29% (4/14)	48% (97/204)	
Unknown	26% (37/140)	8% (4/50)	0%	20% (41/204)	
Reported	21% (30/140)	52% (26/50)	71% (10/14)	32% (66/204)	
♦Mental disorders	17% (5/30)	11% (3/26)	30% (3/10)	17% (11/66)	2.62 [0.83-8.29]
♦Diabetes	43% (13/30)	27% (7/26)	20% (2/10)	33% (22/66)	1.51 [0.68-3.36]
♦Alcohol abuse	67% (20/30)	38% (10/26)	50% (5/10)	53% (35/66)	1.64 [0.90-2.99]
♦Smoking	20% (6/30)	19% (5/26)	10% (1/10)	18% (12/66)	2.19 [0.73-6.52]
♦Drug addiction^5^	17% (5/30)	3% (1/26)	20% (2/10)	12% (8/66)	1.31 [0.32-5.33]
**HIV test**					0.41 [0.22-0.74]**
Not performed/unknown	38% (54/140)	20% (10/50)	0%	31% (64/204)	
Performed	61% (86/140)	80% (40/50)	100% (14/14)	69% (140/204)	
♦Positive	1% (1/86)	5% (2/40)	14% (2/14)	4% (5/140)	4.78 [0.51-44.77]
♦Negative	99% (85/86)	95% (38/40)	86% (12/14)	96% (135/140)	
**Death**	10% (14/140)	40% (20/50)	50% (7/14)	20% (41/204)	4.22 [2.37-7.49]***
Municipality of residence					
Salvador	60% (84/140)	70% (35/50)	79% (11/14)	64% (130/204)	1.20 [0.98-1.47]
Other	40% (56/140)	30% (15/50)	21% (3/14)	36% (74/204)	
**Directly observed treatment**					
Not performed	61% (85/140)	78% (39/50)	64% (9/14)	65% (133/204)	1.24 [1.02-1.50]**
Performed	18% (25/140)	8% (4/50)	36% (5/14)	17%(34/204)	
Unknown	21% (30/140)	14% (7/50)	0%	18% (37/204)	
**Past treatment failure**					
Yes	61% (85/140)	76% (38/50)	100% (14/14)	67% (137/204)	1.34 [1.12-1.60]**
No	18% (26/140)^3^	14% (7/50)	0%	16% (33/204)	
Unknown	21% (29/140)	10% (5/50)	0%	17% (34/204)	
**Past treatment default**					
Yes	37% (52/140)	50% (25/50)	43% (6/14)	41% (83/204)	1.30 [0.94-1.82]
No	42% (59/140)	40% (20/50)	57% (8/14)	43% (87/204)	
Unknown	21% (29/140)	10% (5/50)	0%	17% (34/204)	

**CI:** confidence interval; **MDR-TB:** multidrug-resistant tuberculosis; **XDR-TB:** extensively drug-resistant tuberculosis. ^1^Susceptible to ofloxacin (Ofx), kanamycin (Km), amikacin (Am), and capreomycin (Cm). ^2^Resistant to at least one of the drugs listed previously. ^3^Years. ^4^Patients for which at least one comorbidity was reported. ^5^Abuse of injectable and/or non-injectable drugs. **p-value of <0.01; ***p-value of <0.0001.

Among the comorbidities observed, the most frequent were alcohol abuse and diabetes, followed by smoking and mental disorders. Notably, directly observed treatment (DOT) was implemented in less than half of all cases and in only 3/22 (14%) cases wherein alcohol abuse and/or mental disorders were reported.

While less than 5% of the patients were confirmed to have coinfection with HIV, a significant proportion were never tested (31%; 64/204). Missing HIV test results were found more often among patients with strains sensitive to all second-line anti-TB drugs. The proportion of HIV-coinfected patients was significantly higher among XDR-TB patients than among other patient groups (prevalence ratio: 6.00, 95% CI: 1.09-32.91;p-value=0.039). No information regarding test refusal by the study subjects was available.

TB-related death was the outcome for one-fifth of the patients. Patients with strains exhibiting resistance to at least one second-line anti-TB drug more likely died from TB. All patients coinfected with HIV (2%; 5/204) died; four (4/5, 80%) died from TB.

**FIGURE 1:**
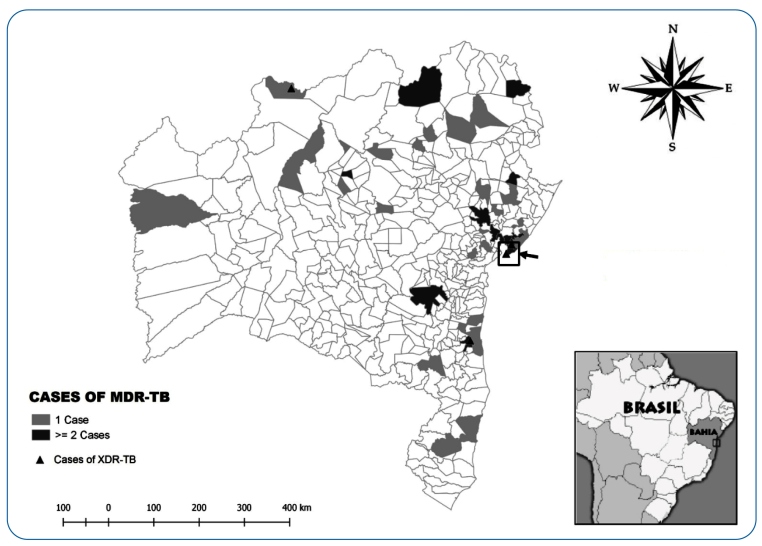
Geographic distribution of multidrug-resistant tuberculosis (MDR-TB) cases in Bahia, Brazil. The location of Salvador is indicated (box with arrow). Municipalities with one case are highlighted in light gray, whereas those with more than one case are highlighted in dark gray. Locations with extensively drug-resistant tuberculosis (XDR-TB) cases are marked with a dark triangle.

The patients resided in 45/417 municipalities in Bahia **(** Figure 1). Two-thirds of the cases were concentrated in Salvador, (130/204), while all other individual municipalities had less than 10% of the total cases. The municipalities of Feira de Santana (10 cases), Paulo Afonso (7 cases), and Jequié (4 cases) had the highest number of cases after Salvador. Almost 80% of the XDR-TB cases were detected in the state capital, compared with that in all other municipalities together, corresponding to a prevalence ratio of 2.09 (95% CI: 0.60-7.24, p-value=0.2464). The remaining analyses were focused on cases originating from Salvador.

Genotyping was successful in 77/130 (59%) of the MDR-TB isolates from Salvador, including 22/35 (63%) of the pre-XDR isolates and 10/11 (91%) of the XDR isolates. We had increased success in genotyping isolates obtained from pre-XDR and XDR patients because they had frequently more than one isolate in the collection. Most of the isolates belonged to the Latin-American Mediterranean family (53/77, 69%) or to the Haarlem family SIT 50 (15%) (Table 2). Similar frequencies were observed in pre-XDR (LAM 68%, H 23%) and XDR (LAM 80%, H 20%) isolates. 

**TABLE 2:** Genotypic profile of MDR-TB isolates from Salvador, Bahia, Brazil (2008-2011).

Family and subfamily	SIT	Octal number	Number of isolates	%
LAM subfamilies				
- LAM9	42	777777607760771	18	13.8
- LAM10-CAMEROON	61	777777743760771	12	9.2
- LAM3	376	376177607760771	12	9.2
	33	776177607760771	9	6.9
- LAM1	20	677777607760771	1	0.8
- Other LAM	59	777777606060771	1	0.8
H3	50	777777777720771	11	8.5
T1	159	777740017760771	4	3.1
U	534	777777607400000	5	3.8
X	119	777776777760771	2	1.5
Other families	Orphan	700000377760771	1	0.8
Not typed	----	----	54	41.5

LAM: Latin American-Mediterranean; H: Haarlem.

**FIGURE 2:**
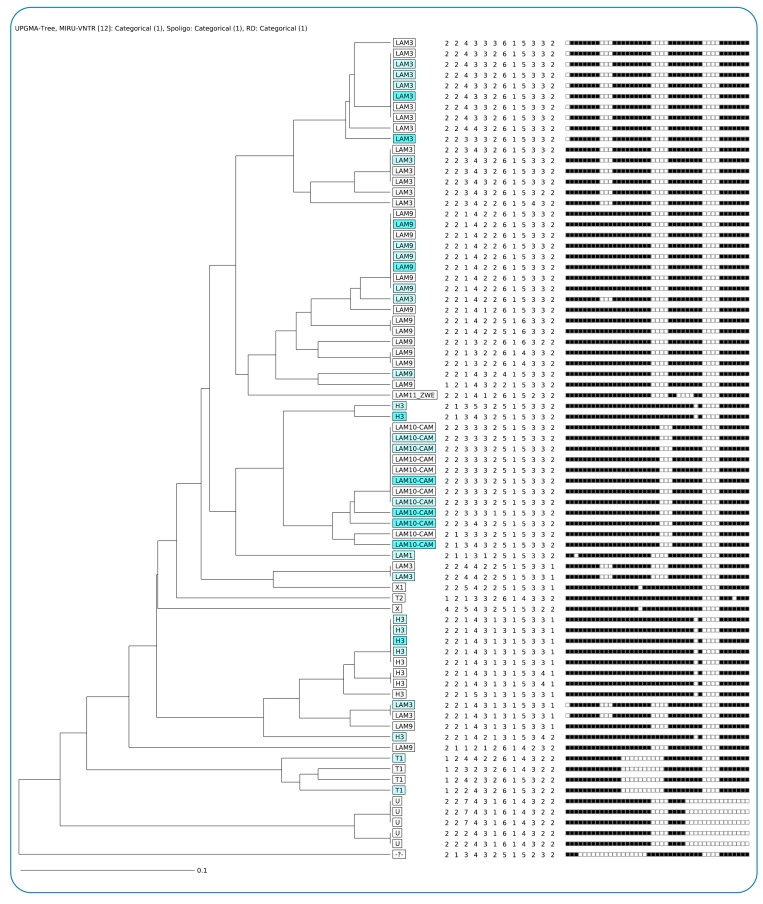
Spoligotype/mycobacterial interspersed repetitive units-variable number of tandem repeats-based unweighted pair-group method using arithmetic avarages dendrogram of the multidrug resistant tuberculosis **(MDR-TB)** isolates from Salvador, Bahia, Brazil. The isolates from pre-extensively drug-resistant tuberculosis **(XDR-TB)** patients are highlighted in pale blue, and those corresponding to XDR-TB patients are highlighted in bright blue.

Twelve clusters of isolates with identical MIRU-VNTR patterns were observed (Figure 2). All clusters with isolates from pre-XDR- and XDR-TB patients (highlighted in this figure) also included isolates sensitive to second-line TB drugs. The largest clusters belonged to the LAM family (SIT 376, 7 isolates; SIT 42, 8 isolates; and SIT 61, 8 isolates). Even though intra-family transmission was suspected in some patients, the genotypic patterns of available isolates that corresponded to these suspected epidemiologically related cases revealed distinct strains. Notably, we found 12 LAM10 isolates grouped within the SIT 61 clade (Cameroon). Isolates from this clade were also identified in two other XDR-TB patients from municipalities outside Salvador (data not shown). This cluster included isolates that corresponded to, respectively, 14% (3/22) and 40% (4/10) of all genotyped pre-XDR-TB and XDR-TB patients. 

**FIGURE 3:**
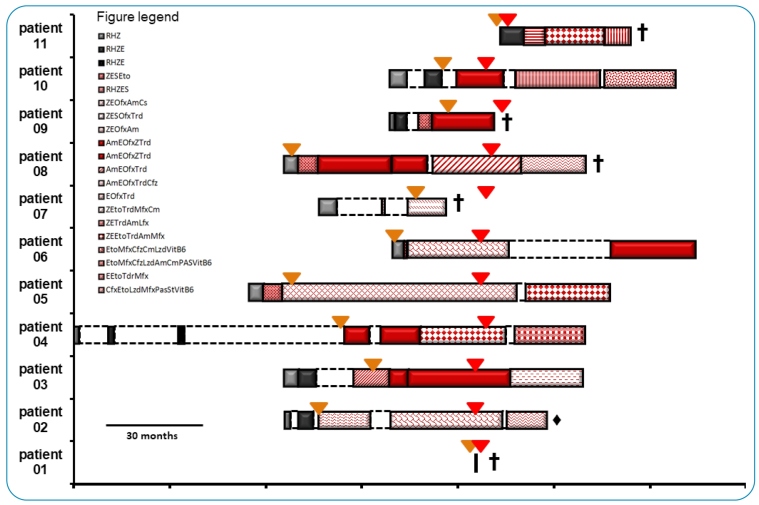
Treatment history of extensively drug-resistant tuberculosis **(XDR-TB)** patients from Salvador, Bahia, Brazil. Bars represent treatment duration for each of the 11 patients studied. The gray boxes represent treatment with first-line anti-TB drugs **(light gray, RHZ; dark gray, RHZE)**, and the red boxes represent treatment with second-line anti-TB drugs (the solid red bar represents the current standard treatment for multidrug-resistant tuberculosis **(MDR-TB)**, personalized regimens are presented in different patterns [see figure legend]). Clear boxes with interrupted outlines represent the time lag between two treatment regimens. The inverted yellow triangles represent the time of MDR-TB diagnosis, and the red ones represent the time of XDR-TB diagnosis (for some of the individuals, XDR-TB diagnosis was available only after patient death, which is represented by a black cross). The black diamond represents treatment default. **R:** rifampin; **H:** isoniazid; **Z:** pyrazinamide; **E:** ethambutol; **S:** streptomycin; **Eto:** ethionamide; **Ofx:** ofloxacin; **Am:** amikacin; **Cs:** cycloserine; **Trd:** terizidone; **Cfz:** clofazimine; **Mfx:** mofloxacin; **Cm:** capreomycin; **Lfx:** levofloxacin; **Lzd:** linezolid; **VitB6:** pyridoxine; **PAS:** para-amino-salicylic acid.

TB-related death was the outcome in 5/11 XDR-TB patients (45%, Figure 3), including two patients with HIV co-infection*.* Patients who underwent more than one course of standard first-line anti-TB treatment were higher (45%; 5/11). The interval between standard treatment completion or interruption and the beginning of a new treatment course varied from the day immediately after completion to approximately 1.8 years (median duration: 83 days, interquartile range: 1-245 days). Two patients (18%, 2/11) were considered TB relapse cases, defined by presenting sputum-positive TB after one registered completion of a standard treatment course^22^. In 6/11 (54%) patients, the treatment regimen was changed before completing the MDR-TB treatment course. In 5/6 (83%) patients, the treatment regimen was changed before XDR-TB diagnosis. Of the six patients, four (4/6, 67%) evolved to cure or completed treatment, while 2/6 (33%) failed treatment or died.

Regarding ethambutol resistance, 9/11 (81%) XDR-TB patients demonstrated resistance while receiving ethambutol as part of their treatment regimen. Six patients with strains that presented resistance to ethambutol continued to receive this drug in their treatment regimens, and three (50%) died. Of the remaining three patients who changed to an ethambutol-free drug regimen, one died due to drug intolerance. The prevalence ratio of death considering these two groups was 1.50 (95% CI: 0.25-8.98, p=0.6569). Two cases of primary XDR-TB died from the disease: one before initiating MDR-TB treatment and the other after being on an MDR-TB regimen for 38 months.

## DISCUSSION

MDR-TB patients in Bahia are predominantly males belonging to black ethnicity and have low education levels. Non-white ethnicity and low education level were previously associated with poorer MDR-TB treatment outcome in Brazil^23^. Male sex and low education levels are associated with TB treatment default^24^ and MDR-TB^25^. Additional resistance to a second-line drug (pre-XDR and XDR combined) was present in one-third of the MDR-TB cases, a feature associated with death. Alcohol use and diabetes were the most highly prevalent comorbidities; both are linked to MDR-TB in other Brazilian studies^26^, and diabetes was previously associated with poorer MDR-TB treatment outcome^27^. Over 10% of the cases reported mental disorders, which may also negatively impact treatment outcomes^23^. 

A minor proportion (18%) of patients received DOT. This finding is in contrast with the reported proportion of DOT among MDR-TB cases in the SINAN database (60.9%)^28^. Patients under DOT have lower rates of unfavorable treatment outcomes, such as treatment default and TB-related death^4^. Patients with comorbidities, such as alcohol use or mental disorders, in Brazil were more likely to receive DOT^28^, yet this was not the case in our study population.

The proportion of HIV-positive MDR-TB cases herein was low. This finding is similar to results obtained by Matos et al.^8^, yet it is in contrast to those of previous reports in Brazil detailing an association between MDR-TB and HIV coinfection^29^. A significant proportion of MDR-TB cases were not tested for HIV, which directly contradicts current Brazilian TB management guidelines^30^, as well as the nationwide conduct reported in other settings. Nonetheless, the proportion of patients who were not tested remains similar to the national average^7^. The cause(s) for this discrepancy must be elucidated. One hypothesis is that MDR-TB diagnosis may have occurred too late, i.e., close to death, which reduces timeliness in conducting HIV diagnosis. TB-HIV coinfection is associated with progressively more resistant forms of TB^31^, as well as increased risk of adverse events and death, especially for patients not undergoing antiretroviral treatment^32^. HIV coinfection was associated with death among MDR-TB patients in Brazil^23^. All HIV-positive MDR-TB patients in our setting died. 

MDR-TB cases were reported in 11% of the municipalities of Bahia, two-thirds of case notifications originated from residents in the municipality of Salvador. By contrast, a nationwide data registry of TB cases indicated the presence of disease in more than half of all Brazilian cities^11^. We hypothesize that MDR-TB is inefficiently detected in the municipalities throughout Bahia compared with the state capital. These municipalities may indeed be more affected by insufficient training of healthcare teams, high mobility of healthcare personnel, excessive workloads in people responsible for healthcare management, and reduced governmental healthcare funding^33^. Given this limitation, we opted to focus our analyses on the samples obtained from Salvador.

While therapeutic success rates among MDR-TB patients have improved in southeastern Brazil, poor treatment outcomes continue to persist in the northeastern region, where Bahia is located^27^. Retreatment and treatment failure were frequent among the study subjects, and both are associated with an increased risk of adverse events and the development of drug-resistant TB^27^. In 2021, only 44% of all patients previously treated for TB underwent a drug susceptibility test in Brazil; the proportion of drug susceptibility tests in the same subpopulation in Bahia is even smaller (37%)^7^. Furthermore, primary XDR-TB occurrence in our settings was related to inadequate treatment and death. The fact that drug susceptibility testing is performed less consistently in the Brazilian northeast raises additional concerns regarding the potential risk of provoking a more worrisome scenario in the coming years. In the early 2000s, TB strains resistant to at least four drugs represented less than 2% of all drug-resistant strains in Salvador^8^. Notably, early detection of primary resistance to rifampicin has been facilitated since the introduction of the GeneXpert MTB/RIF assay as a rapid test for TB diagnosis, in 2015^7^. We therefore highlight the importance of performing drug-susceptibility testing as essential to the design of effective treatment regimens.

Similar to previous studies from Brazil^34^ ^-^ ^37^, we found that LAM9 SIT 42 and Haarlem H3 SIT 50 were frequent among the strains obtained from MDR-TB patients in Salvador. Two previous studies reported LAM10 SIT 61 (Cameroon)^34^ ^,^ ^36^, albeit a very low prevalence. This clade originates from Africa and has been associated with drug resistance in Cameroon^38^. Its occurrence in our setting may be related to recent transmission due to migration from Africa, but other evidence links the occurrence of African TB strains in Brazil to the slave trade. In fact, evidence regarding the detection of East-African-Indian strains in northeastern Brazil suggests a common ancestry with strains from Eastern Africa^39^.

To the best of our knowledge, the present study is the first to report XDR-TB detection in Bahia, Brazil. XDR-TB represented 7% of the MDR-TB cases studied, a proportion similar to that reported in other Brazilian states^34^. Gayoso et al.^24^ reported that XDR-TB patients registered in the Brazilian MDR-TB surveillance system from January 2005 to December 2012 represented 1.8% of all MDR-TB patients nationwide.

Discrepancies in the treatment regimens adopted and individual drug susceptibility profiles clearly indicate that laboratory results should be made available in a timelier manner for effective management of XDR-TB patients in our setting. The delay in XDR-TB treatment initiation when using conventional drug susceptibility testing is a recognized issue for disease management. A study conducted in a contemporaneous cohort from South Africa reported a medium delay of 65 days from sputum acquisition to treatment initiation^40^ ^-^ ^41^. The use of only partially effective drug regimens in the absence of information on a patient’s drug susceptibility profile may favor the development of further resistance^5^. It is also concerning that primary MDR/XDR-TB drug resistance may not always be recognized, leading to an ineffective initial treatment regimen and possibly undetected disease transmission. 

Our study is limited in some respects: 1) We could conduct only 12-loci evaluations, which is insufficient to reliably detect more recent MDR/XDR-TB transmission. Whole-genome sequencing is a reliable technique to investigate TB transmission^42^ and may help clarify transmission dynamics in our settings. Nonetheless, classical epidemiologic data strengthen our hypotheses. 2) A high degree of incompleteness was observed for some important variables in the secondary databases available. During the study period (and continuing to the present day), a considerable proportion of MDR-TB resistance remains undiagnosed because of the lack of culture testing not being solicited. We emphasize that all patients who received MDR-TB diagnosis in Bahia during this period were referred to the reference hospital, and sputum cultures were appropriately performed by the state reference laboratory and stored in their collection for surveillance purposes. 

Our data raise concerns that the active transmission of MDR/XDR-TB may occur at a higher rate than that previously estimated. It is also possible that biological differences among the circulating strains in our setting may impact the genetic distribution of *M. tuberculosis* and consequently favor resistance within some clades. In this regard, the Cameroon family is described as presenting genetic features that may cause alterations in pathogenic behavior, e.g., changes in metabolic pathways due to insertion elements (IS) in genes involved in physiological processes^38^. The possibility that such changes may confer a selective advantage deserves further investigation.

## CONCLUSION

MDR-TB presents a challenge to TB control efforts in our setting, as affected individuals frequently present alcohol and substance abuse, as well as mental disorders, which may hinder treatment adherence. Moreover, the laboratory diagnosis of HIV coinfection and MDR/XDR TB may not be performed in a timely manner, which hinders the proper management of patients, potentially increasing TB death rates. Our genetic findings suggest that the transmission of MDR/XDR-TB strains may occur at higher rates than previously suspected, which raises further concern regarding the control measures that should be put in place to mitigate the increasing MDR seen in our setting.
